# Low serum adiponectin level is associated with better physical health-related quality of life in chronic kidney disease

**DOI:** 10.1038/s41598-021-90339-8

**Published:** 2021-05-25

**Authors:** Ji Hye Kim, Ji Min Han, Hyang Kim, Kyu-Beck Lee, Wookyung Chung, Yong-Soo Kim, Sue K. Park, Dong Wan Chae, Curie Ahn, Kook-Hwan Oh, Young Youl Hyun, KNOW-CKD Study Group

**Affiliations:** 1 Department of Internal Medicine, Kangbuk Samsung Hospital, Sungkyunkwan University School of Medicine, 29 Saemunan-ro, Jongno-Gu, Seoul, 03181 Republic of Korea; 2 Jung Jaemyun Internal Medicine Clinic, Seoul, Republic of Korea; 3 Department of Internal Medicine, Gachon University, Gil Medical Center, Incheon, Republic of Korea; 4 Department of Internal Medicine, The Catholic University of Korea, Seoul St. Mary’s Hospital, Seoul, Republic of Korea; 5 Department of Preventive Medicine, Seoul National University College of Medicine, Seoul, Republic of Korea; 6 Department of Internal Medicine, Seoul National University Bundang Hospital, Seongnam, Gyeonggi-do Republic of Korea; 7 Department of Internal Medicine, Seoul National University College of Medicine, Seoul, Republic of Korea

**Keywords:** Medical research, Nephrology

## Abstract

Hyperadiponectemia is paradoxically associated with renal disease progression and mortality in chronic kidney disease (CKD). Its association with health-related quality of life (HR-QOL) is unknown. This study aimed to verify the association between adiponectin and HR-QOL in Korean pre-dialysis CKD cohort. This cross-sectional study analyzed 1551 pre-dialysis CKD patients from KNOW-CKD (KoreaN Cohort Study for Outcome in Patients With Chronic Kidney Disease). Participants were categorized into three tertiles (T1–T3) according to adiponectin levels. HR-QOL was assessed using SF-36. High physical component summary (PCS) and mental component summary (MCS) were defined as highest quartile of each score. Multivariate logistic regression was used to analyze odds ratio (OR) and 95% confidence interval (CI) for high PCS and MCS. Prevalence of high PCS were 33.3%, 27.5%, and 17.0% and that of high MCS were 31.7%, 24.8%, and 21.3% for T1, T2, and T3 (both p for trend < 0.001). The adjusted OR [95% CI] of T1 and T2 in reference to T3 were 1.56 [1.09–2.23] and 1.19 [0.85–1.68] for high PCS and 1.19 [0.85–1.68] and 0.94 [0.68–1.29] for high MCS. Serum adiponectin level was inversely associated with physical HR-QOL in Korean pre-dialysis CKD patients. This relationship was independent of various cardiovascular risk factors.

## Introduction

Adiponectin is a 30-kDa, 244 amino acid polypeptide hormone secreted by adipocytes. It is the most abundant adipokine with insulin-sensitizing, anti-inflammatory, and anti-atherogenic properties^1^. Several epidemiological studies have shown that hypoadiponectinemia is associated with increased metabolic and cardiovascular disease prevalence^2–4^. On the other hand, recent studies have shown that hyperadiponectinemia is associated with high mortality^5,6^. Moreover, there are conflicting results as to whether adiponectin plays a protective role in kidney disease. Some studies have shown that hypoadiponectinemia in CKD is associated with insulin resistance, inflammation, and increased cardiovascular risks^7,8^. However, majority of the studies have shown that hyperadiponectinemia is paradoxically a risk factor for renal disease progression and high mortality in CKD^9,10^.

There is a growing interest in evaluating the health-related quality of life (HR-QOL) in CKD patients. HR-QOL is defined as an individual or group’s perceived physical and mental health over the duration of a disease^11^. Since HR-QOL is relative to the severity of the patient’s perceived symptoms, HR-QOL itself can be a clinical outcome indicator^12^. CKD is a disease with particularly low HR-QOL. In CKD patients, HR-QOL decreases after initiation of hemodialysis or peritoneal dialysis and increases after kidney transplantation^13^. Low HR-QOL is a risk factor for CKD progression, cardiovascular disease prevalence, hospitalization, and mortality^14–16^.

In previous studies, high adiponectin level has been associated with malnutrition or anemia, which can affect HR-QOL^17,18^. Therefore, we hypothesized that adiponectin would affect HR-QOL in CKD. Despite enormous interest in adiponectin and HR-QOL, the relationship between serum adiponectin level and HR-QOL is unknown in CKD. Therefore, this cross-sectional study was conducted to investigate the association between serum adiponectin level and HR-QOL in the Korean pre-dialysis CKD cohort.

## Methods

### Study design and population

**Figure 1:**
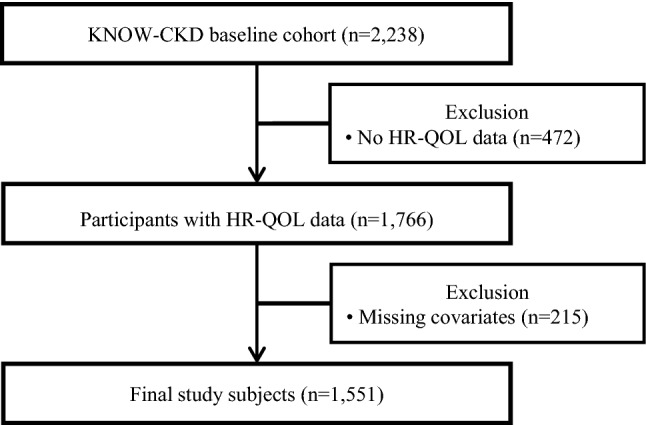
Algorithm for study patient selection from the KNOW-CKD cohort. HR-QOL, health-related quality of life.

This cross-sectional study was designed to examine the association between serum adiponectin level and HR-QOL in pre-dialysis CKD patients. ‘KoreaN Cohort Study for Outcome in Patients With Chronic Kidney Disease’ (KNOW-CKD) is an ongoing multicenter, prospective study which includes pre-dialysis CKD patients from nine nephrology centers of major university hospitals in Korea. A total of 2238 adults between 20 and 75 years of age were enrolled from ‘KNOW-CKD’ study from 2011 to 2016. Among them, 472 and 215 participants were excluded due to missing HR-QOL information and missing data for variables of interest. Finally, 1551 patients were included for analysis in this study (Fig. 1).

The study was conducted in accordance with the principles of the Declaration of Helsinki and supervised by the Korea Centers for Disease Control and Prevention. All study participants were provided with written consent forms. ‘KNOW-CKD’ was approved by the Institutional Review Boards of Kangbuk Samsung Medical Center, Seoul National University Hospital, Seoul St. Mary’s Hospital, Severance Hospital, Gil Hospital, Eulji General Hospital, Chonnam National University Hospital and Pusan Paik Hospital in 2011. A detailed protocol of this study has been previously published^19^.

### Clinical and laboratory measurements

All clinical and laboratory measurement were collected from patients on their initial visit to each hospital. Sociodemographic data, including education, marital status, and income, underlying medical history, medication, and lifestyle patterns, including cigarette smoking and alcohol consumption were collected using self-reported questionnaires with the assistance of a trained staff.

Blood samples were collected after at least 8 h of fasting. Serum adiponectin level was measured using a commercial enzyme-linked immunosorbent assay (ELISA) kit (Adipogen Corp., San Diego, CA, USA). This method had intra- and inter-assay coefficients of variations of ≤ 3.84 and ≤ 5.50%, respectively. Random urine samples were collected midstream for the urine protein-to-creatinine ratio (PCR) measurement. Serum creatinine, cystatin C, and 25-(OH)-vitamin D were measured at the Lab Genomics, Seoul, Republic of Korea. Serum creatinine was measured using an isotope dilution mass spectrometry (IDMS)-traceable method. Other biochemical analyses were conducted in each participating hospital’s laboratory. Estimated glomerular filtration rate (eGFR) was calculated using the Chronic Kidney Disease Epidemiology Collaboration (CKD-EPI) equation.

Blood pressure (BP) was measured by a trained nurse using an electronic sphygmomanometer after 5 min of rest in the sitting position. Hypertension was defined as: (a) systolic blood pressure (SBP) > 140 mmHg or diastolic blood pressure > 90 mmHg or (b) previous diagnosis of hypertension. Diabetes mellitus (DM) was defined as: (a) fasting serum glucose > 126 mg/dL or (b) previous diagnosis of DM.

### HR-QOL assessment

Kidney Disease Quality of Life-Short Form (KDQOL-SF) instrument was used to assess comprehensive HR-QOL in CKD patients^20^. The KDQOL-SF contains a 36-item short form survey (SF-36) that includes a total of 36 items from each of the four domains of physical and mental health measurements. The Korean versions of KDQOL-SF and SF-36, which were verified as valid HR-QOL instruments in previous studies, were used in this study^21,22^. The questionnaires were completed with the aid of trained personnel if help was needed. The SF-36 is composed of two dimensions: physical component summary (PCS) and mental component summary (MCS). PCS includes the following four domains: (1) physical function, (2) role limitations due to physical problems, (3) body pain, and (4) general physical health. MCS includes the following four domains: (1) vitality, (2) limitation due to emotional problems, (3) social function, and (4) general mental health. Response to each PCS and MCS dimension was converted to the SF-36 equivalent score ranging from 0 to 100. Higher numerical scores indicate better HR-QOL or less impairment. In this study, the PCS and MCS scores were the primary outcomes of interest. High PCS and MCS were defined as the highest quartile score of each dimension.

### Statistical analysis

Participants were categorized into three groups (T1, T2, and T3) according to serum adiponectin tertile. Continuous variables were expressed as mean ± standard deviation (SD) or median and interquartile range. Continuous variables were analyzed using the Kruskal–Wallis test or one-way analysis of variance. Categorical variables were expressed as percentages. Comparison of categorical variables between each adiponectin tertile group was performed using the Chi-square test.

We constructed generalized linear model and plotted adjusted predictions of PCS and MCS according to serum adiponectin tertile using the Stata command, marginsplot. We used multivariate logistic regression analysis to estimate the odds ratio (OR) and 95% confidence interval (CI) for the high PCS and MCS. These regression models were adjusted for age, sex, body mass index (BMI), marriage, economy, education, systolic BP, DM, dyslipidemia, cardiovascular disease, eGFR, urine PCR, C-reactive protein (CRP), hemoglobin, current smoking status, alcohol intake, and physical activity measured as metabolic equivalent (MET).

All statistical analyses were performed using the Stata version 15.0 (StataCorp LP, College Station, TX, USA). Two-sided P-values < 0.05 were considered statistically significant.

## Results

**Figure 2:**
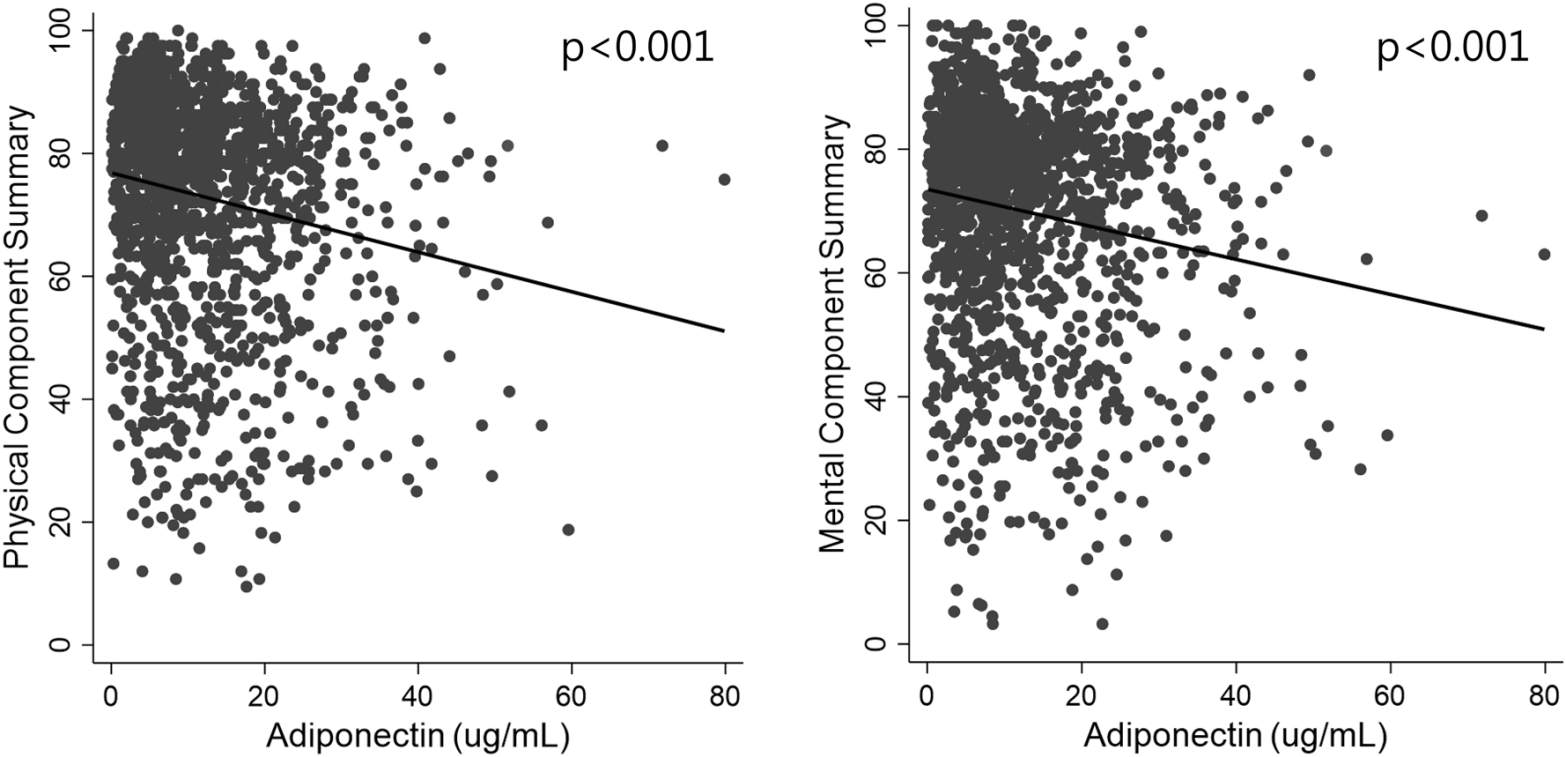
Scatter plot of the HR-QOL scores versus serum adiponectin (**A**; physical component summary, **B**; mental component summary).

The mean age of the overall study population was 52.5 ± 12.4 years, and 61.25% of them were male. The mean serum adiponectin level was 72.87 ± 18.26 ug/mL, and the range was between 0.12 and 79.89 ug/mL. In comparison of the baseline characteristics of the study population according to adiponectin tertiles, the higher tertile groups had higher percentage of females and urine PCR levels, and lower BMI, eGFR, CRP, hemoglobin, and serum albumin. In the sociodemographic and lifestyle pattern domains, the higher tertile groups had lower percentage of those with high education, high income, current smokers, and those who consumed alcohol more than twice a week. Furthermore, the PCS score was lower and the percentage of those with high PCS was also lower in the higher adiponectin tertile groups. Similar trend was seen with MCS, as the mean MCS score was lower and the percentage of those with high MCS was also lower in the higher adiponectin tertile groups (Table 1). The scatter plot of each PCS and MCS scores versus serum adiponectin level shows their inverse correlations (Fig. 2).

**Table 1:** Baseline characteristic of participants according to serum adiponectin tertile.

	Adiponectin tertile				p for trend
	Total (0.12–79.89)	T1 (0.12–6.54)	T2 (6.55–13.71)	T3 (13.74–79.89)	
Total number of patients	1551	517	517	517	
Adiponectin, ug/mL	72.87 ± 18.26	3.74 ± 1.80	9.80 ± 2.17	23.28 ± 8.92	
Age, years	52.5 ± 12.4	51.4 ± 12.2	53.2 ± 12.1	53 ± 12.7	0.039
Male sex	950 (61.3)	389 (75.2)	312 (60.4)	249 (48.2)	< 0.001
BMI, kg/m^2^	24.5 ± 3.4	25.4 ± 3.1	24.6 ± 3.4	23.4 ± 3.4	< 0.001
Systolic BP, mmHg	128 ± 16	128 ± 16	130 ± 18	129 ± 16	0.06
Diastolic BP, mmHg	77 ± 11	77 ± 11	76 ± 11	78 ± 11	0.404
eGFR, mL/min/1.73 m^2^	54.1 ± 31.5	63.5 ± 31.6	54.5 ± 29.1	44.3 ± 30.8	< 0.001
Urine PCR, g/g	0.5 (0.1**–**1.5)	0.4 (0.1**–**1)	0.5 (0.1**–**1.4)	0.7 (0.2**–**2.3)	< 0.001
CRP, mg/dL	0.6 (0.2**–**1.6)	0.8 (0.3**–**2)	0.6 (0.2**–**1.5)	0.5 (0.2**–**1.4)	< 0.001
Hemoglobin, g/dL	12.9 ± 2	13.7 ± 2	13 ± 1.8	11.9 ± 1.7	< 0.001
Serum albumin, g/dL	4.2 ± 0.4	4.3 ± 0.3	4.2 ± 0.4	4.0 ± 0.5	< 0.001
High education^a^	682 (44.0)	267 (51.6)	227 (43.9)	188 (36.4)	< 0.001
Diabetes	497 (32.0)	174 (33.7)	152 (29.4)	171 (33.1)	0.842
Dyslipidemia	831 (53.6)	284 (54.9)	286 (55.3)	261 (50.5)	0.152
Cardiovascular disease	200 (12.9)	64 (12.4)	75 (14.5)	61 (11.8)	0.781
Married	1286 (82.9)	423 (81.8)	443 (85.7)	420 (81.2)	0.804
High income^b^	367 (23.7)	132 (25.5)	133 (25.7)	102 (19.7)	0.028
Current smoker	255 (16.4)	109 (21.1)	83 (16.1)	63 (12.2)	< 0.001
Alcohol intake^c^	218 (14.1)	87 (16.8)	75 (14.5)	56 (10.8)	0.006
Physical activity, MET min/wk	1188 (264**–**2754)	1332 (297**–**2853)	1188 (297**–**2613)	990 (198**–**2754)	0.855
PCS	72.9 ± 18.3	76.1 ± 16.9	73.9 ± 17.8	68.7 ± 19.3	< 0.001
MCS	70 ± 18.1	72.5 ± 17.4	71.2 ± 17	66.4 ± 19.2	< 0.001
High PCS	402 (25.9)	172 (33.3)	142 (27.5)	88 (17.0)	< 0.001
High MCS	402 (25.9)	164 (31.7)	128 (24.8)	110 (21.3)	< 0.001

Values for categorical variables are reported as number (percentage) and for continuous variables as mean ± standard deviation or median (interquartile range).

The p values are for the Kruskal–Wallis tests or one-way analysis of variance for continuous variables and Chi-square tests for categorical variables.

*PCS* physical component summary; *MCS* mental component summary; *BMI* body mass index; *SBP* systolic blood pressure; *DBP* diastolic blood pressure; *LDL* low-density lipoprotein; *CRP* C-reactive protein; *eGFR* estimated glomerular filtration rate; *PCR* protein to creatinine ratio; *MET* metabolic equivalent.

^a^College diploma or more.

^b^More than 4.5 million won per month.

^c^Alcohol intake frequency ≥ 2/week.

**Figure 3:**
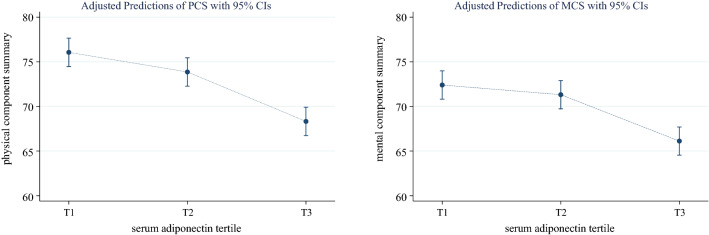
Adjusted predictions of physical component summary and mental component summary according to serum adiponectin tertile. Adjusted for age, sex, body mass index, marriage, economy, education, systolic blood pressure, diabetes, dyslipidemia, cardiovascular disease, estimated glomerular filtration rate, urine protein to creatinine ratio, C-reactive protein, hemoglobin, albumin current smoking, alcohol, and physical activity.

Figure 3 shows the adjusted predictions of PCS and MCS scores according to serum adiponectin tertiles, and both scores were lower in T3. In the multivariate logistic regression analysis, lower adiponectin levels were associated with high PCS scores. As shown in Table 2, the OR (95% CI) for high PCS was 1.44 (1.03–2.01) and 1.56 (1.09–2.23) in T2 and T1, respectively, compared with T3 (Reference). However, serum adiponectin level was not associated with high MCS. (Table 3).

**Table 2:** Multivariate analysis of the association between serum adiponectin tertile and high physical component summary (PCS).

Adiponectin tertile	Unadjusted model		Model 1		Model 2	
	OR [95% CI]	p value	OR [95% CI]	P value	OR [95% CI]	p value
T1	2.43 [1.81–3.26]	< 0.001	1.98 [1.44–2.73]	< 0.001	1.56 [1.09–2.23]	0.015
T2	1.85 [1.37–2.49]	< 0.001	1.65 [1.20–2.25]	0.002	1.44 [1.03–2.01]	0.033
T3	Reference		Reference		Reference	

Model 1: Adjusted for age, sex, body mass index, marriage, economy, education, systolic blood pressure, diabetes, dyslipidemia, cardiovascular disease, estimated glomerular filtration rate, urine protein to creatinine ratio, C-reactive protein, hemoglobin, and albumin.

Model 2: Adjusted for Model 1 + current smoking, alcohol, and physical activity.

**Table 3:** Multivariate analysis of the association between serum adiponectin tertile and high mental component summary (MCS).

Adiponectin tertile	Unadjusted model		Model 1		Model 2	
	OR [95% CI]	p value	OR [95% CI]	P value	OR [95% CI]	p value
T1	1.72 [1.30–2.28]	< 0.001	1.41 [1.04–1.92]	0.028	1.19 [0.85–1.68]	0.312
T2	1.22 [0.91–1.63]	0.184	1.04 [0.77–1.41]	0.795	0.94 [0.68–1.29]	0.694
T3	Reference		Reference		Reference	

Model 1: Adjusted for age, sex, body mass index, marriage, economy, education, systolic blood pressure, diabetes, dyslipidemia, cardiovascular disease, estimated glomerular filtration rate, urine protein to creatinine ratio, C-reactive protein, hemoglobin, and albumin.

Model 2: Adjusted for Model 1 + current smoking, alcohol, and physical activity.

In the subgroup analyses of clinically relevant groups, the association between serum adiponectin level and high PCS was similar across all subgroups with no significant interactions between sex (female vs. male), age (< 55 vs. ≥ 55 years), BMI (< 23.5 vs. ≥ 23.5 kg/m^2^), diabetes (no vs. yes), proteinuria (< 1.0 vs. ≥ 1.0 g/day), and CRP (< 0.5 vs. ≥ 0.5 mg/dL). Although the association was modified by the eGFR level (< 45 vs. ≥ 45 mL/min/1.73 m^2^), the trend was similar (Table 4).

**Table 4:** Association between serum adiponectin tertile and high PCS in clinically relevant subgroups.

	Adiponectin tertile			
	T1	T2	T3	p for interaction
	OR (95% CI)	OR (95% CI)	OR (95% CI)	
**Sex**				0.591
Female (n = 601)	1.25 [0.67–2.34]	1.40 [0.83–2.35]	Reference	
Male (n = 950)	1.69 [1.07–2.68]	1.45 [0.93–2.28]	Reference	
**Age, years**				0.678
< 55 (n = 820)	1.68 [1.03–2.73]	1.34 [0.85–2.11]	Reference	
≥ 55 (n = 731)	1.38 [0.79–2.40]	1.59 [0.96–2.65]	Reference	
**BMI, kg/m**^**2**^				0.564
< 23.5 (n = 605)	1.15 [0.65–2.04]	1.08 [0.67–1.76]	Reference	
≥ 23.5 (n = 946)	1.96 [1.17–3.27]	1.81 [1.10–2.99]	Reference	
**Diabetes**				0.532
Diabetes (−) (n = 1054)	1.80 [1.18–2.74]	1.66 [1.13–2.45]	Reference	
Diabetes ( +) (n = 497)	1.11 [0.55–2.26]	0.87 [0.43–1.76]	Reference	
**eGFR, mL/min/1.73 m**^**2**^				0.029
< 45 (n = 734)	2.73 [1.54–4.85]	2.22 [1.30–3.79]	Reference	
≥ 45 (n = 817)	1.02 [0.64–1.64]	1.04 [0.67–1.63]	Reference	
**Urine protein/creatinine, g/g**				0.925
< 1.0 (n = 1039)	1.74 [1.12–2.70]	1.50 [0.99–2.26]	Reference	
≥ 1.0 (n = 512)	1.28 [0.66–2.46]	1.23 [0.67–2.26]	Reference	
**CRP, mg/dL**				0.452
< 0.5 (n = 632)	1.50 [0.87–2.60]	1.22 [0.75–1.98]	Reference	
≥ 0.5 (n = 919)	1.56 [0.95–2.56]	1.60 [0.99–2.58]	Reference	

*BMI* body mass index; *eGFR* estimated glomerular filtration rate; *CRP* C-reactive protein.

## Discussion

In this cross-sectional study, we analyzed the association between serum adiponectin level and HR-QOL in the Korean pre-dialysis CKD patients. Patients with lower serum adiponectin level had higher PCS and MCS scores than those with high serum adiponectin level. According to multivariate logistic regression analyses, low serum adiponectin level was significantly and independently associated with high PCS. This association was preserved in clinically relevant subgroups. However, there was no statistically significant association between serum adiponectin level and high MCS.

Adiponectin is a 30-kDa, 244 amino acid polypeptide hormone released by adipocytes, which exists as low, medium, and high molecular weight proteins^23^. It has insulin-sensitizing, anti-inflammatory, and anti-atherogenic functions towards different cell types. Several previous studies have reported that higher serum adiponectin level has a protective function against metabolic and cardiovascular diseases, as it is associated with higher HDL-cholesterol levels and lower DM and cardiovascular disease risks^1,2,9,24^. However, in CKD patients, higher serum adiponectin level is paradoxically associated with proteinuria, CKD progression, and all-cause mortality^9,10,23,25,26^.

Previous studies have investigated the clinical implication of HR-QOL in CKD^13–15,27^. In patients with end-stage renal disease, low HR-QOL was generally associated with higher risks of mortality and hospitalization^16,28,29^. According to a study that assessed the HR-QOL in 3837 CKD patients, low HR-QOL was associated with higher risk of cardiovascular event and mortality, but not with CKD progression^15^. On the other hand, according to a Korean study that assessed the impact of HR-QOL in CKD progression, lower PCS score was associated with increased CKD progression risk^14^.

To our knowledge, there is not yet a study that has assessed the association between serum adiponectin level and HR-QOL in pre-dialysis CKD patients. According to our study, low serum adiponectin level was associated with better physical HR-QOL. The mechanism underlying this association is unknown; however, we can suggest several explanations for our finding.

First, previous studies have reported association between high adiponectin levels and malnutrition in pre-dialysis CKD patients^17,30,31^. A large percentage of CKD patients are in a ‘malnutrition-inflammation’ status caused by reduced caloric intake due to symptoms of uremia, reduced absorption of nutrients, cachexia, and persistent inflammatory status. In CKD, protein-energy wasting is caused by a complex interaction of persistent inflammation, malnutrition, acidosis status, and increased catabolism, resulting in sarcopenia^32^. It is assumed that serum adiponectin levels are increased to counteract this ‘malnutrition-inflammation’ status^33^. Furthermore, according to a Korean study, mildly obese CKD patients were more likely to be in a state of adequate nutrition without inflammation than those with low BMI and total body fat^34^. Consistent with the above finding, results from our study showed that the highest adiponectin tertile group (T3) had significantly lower BMI and serum albumin level than the lower adiponectin tertile groups (T1,T2). The combined results of reduced muscle mass, body fat mass, and symptoms of fatigue caused by malnutrition would have contributed to limitations in physical activity, as reflected by lower PCS scores in the high adiponectin tertile group.

Second, serum adiponectin exists mainly as two types of hexamers: as a low molecular weight complex (LMW) and as a high molecular weight (HMW) complex consisting of 12–18 subunits^35^. The ratio between the two complexes (HMW to LMW complex) determines its insulin-sensitizing activity^36^. In CKD patients, the altered ratios of the adiponectin complexes result in a decreased insulin-sensitizing activity. Subsequently, this leads to an increased insulin resistance that ultimately increases muscle proteolysis resulting in an increased skeletal muscle wasting and weakness associated with poor physical QOL^36^.

Third, previous studies have shown that high serum adiponectin levels were associated with anemia in CKD patients^18,37^. Anemia is associated with low HR-QOL, especially physical activity impairment, due to its symptoms, including fatigue, dizziness, dyspnea as well as being in an inflammatory state^38–40^. Consistent results were found in our study as hemoglobin levels were significantly lower in the high adiponectin tertile group than in the lower tertile groups.

It is unclear why low adiponectin level was only associated with high PCS and not high MCS. We hypothesize the mechanism behind this finding as the following. Previous studies have reported that low serum adiponectin level was associated with various mental disorders, including insomnia and depression, and neurodegenerative disorders, including Alzheimer’s disease^41–43^. Insomnia, depression, and Alzheimer’s disease are comorbidities that can affect the MCS score. As CKD patients are in a relatively hyperadiponectemia state, the increased adiponectin level may have had neuroprotective effects and influenced the association between adiponectin and mental HR-QOL.

There are a few limitations of this study that include the following. First, as this is a cross-sectional study, we cannot determine the causal relationship between serum adiponectin level and HR-QOL. Further longitudinal studies would be helpful to verify the causality. Second, adiponectin level and HR-QOL are both affected by body compositions, such as adiposity and muscle mass. We included BMI in the analytical model, but unfortunately, we have no data on body composition analysis.

Despite these limitations, our study also has strengths. The KNOW-CKD is a well-organized, multi-center, prospective cohort study that employed a very strict and uniform protocol to collect and record data. In addition, we included various factors in the analysis, including sociodemographic, lifestyle information, and comorbid status, which could affect the HR-QOL.

In conclusion, low adiponectin levels were associated with better physical HR-QOL in pre-dialysis CKD patients, and this association was independent of various metabolic and cardiovascular factors. This study suggests the possible role of measuring serum adiponectin level in evaluating CKD patients. More attention should be given to the HR-QOL and prognosis of patients with low serum adiponectin level. Further studies, including longitudinal studies, are needed to confirm the relationship between serum adiponectin level and HR-QOL and its clinical significance.

## References

[1] Turer AT, Scherer PE (2012). Adiponectin: Mechanistic insights and clinical implications. Diabetologia.

[2] Van de Voorde J, Pauwels B, Boydens C, Decaluwe K (2013). Adipocytokines in relation to cardiovascular disease. Metabolism.

[3] Van Berendoncks AM, Garnier A, Ventura-Clapier R, Conraads VM (2013). Adiponectin: Key role and potential target to reverse energy wasting in chronic heart failure. Heart Fail. Rev..

[4] Wang Y (2018). Plasma adiponectin levels and type 2 diabetes risk: A nested case-control study in a Chinese population and an updated meta-analysis. Sci. Rep..

[5] Menzaghi C, Trischitta V (2018). The adiponectin paradox for all-cause and cardiovascular mortality. Diabetes.

[6] Scarale MG, Fontana A, Trischitta V, Copetti M, Menzaghi C (2018). Circulating adiponectin levels are paradoxically associated with mortality rate: A systematic review and meta-analysis. J. Clin. Endocrinol. Metab..

[7] Becker B (2005). Renal insulin resistance syndrome, adiponectin and cardiovascular events in patients with kidney disease: The mild and moderate kidney disease study. J. Am. Soc. Nephrol..

[8] Zoccali C (2002). Adiponectin, metabolic risk factors, and cardiovascular events among patients with end-stage renal disease. J. Am. Soc. Nephrol..

[9] Lo MM, Mitsnefes M (2012). Adiponectin, cardiovascular disease, chronic kidney disease: Emerging data on complex interactions. Pediatr. Nephrol..

[10] Heidari M, Nasri P, Nasri H (2015). Adiponectin and chronic kidney disease; a review on recent findings. J. Nephropharmacol..

[11] Centers for Disease Control and Prevention. *HRQOL Concepts*, https://www.cdc.gov/hrqol/concept.htm (2018).

[12] Yapa HE, Purtell L, Chambers S, Bonner A (2019). The relationship between chronic kidney disease, symptoms and health-related quality of life: A systematic review. J. Ren Care.

[13] Avramovic M, Stefanovic V (2012). Health-related quality of life in different stages of renal failure. Artif. Organs.

[14] Oh TR (2019). Association between health related quality of life and progression of chronic kidney disease. Sci. Rep..

[15] Porter AC (2016). Predictors and outcomes of health-related quality of life in adults with CKD. Clin. J. Am. Soc. Nephrol..

[16] Mapes DL (2003). Health-related quality of life as a predictor of mortality and hospitalization: The Dialysis Outcomes and Practice Patterns Study (DOPPS). Kidney Int..

[17] Hyun YY (2017). Serum adiponectin and protein-energy wasting in predialysis chronic kidney disease. Nutrition.

[18] Kim H (2018). High serum adiponectin is associated with anemia development in chronic kidney disease: The results from the KNOW-CKD study. Cytokine.

[19] Oh KH (2014). KNOW-CKD (KoreaN cohort study for Outcome in patients With Chronic Kidney Disease): Design and methods. BMC Nephrol..

[20] Hays RD, Kallich JD, Mapes DL, Coons SJ, Carter WB (1994). Development of the kidney disease quality of life (KDQOL) instrument. Qual. Life Res..

[21] Park HJ (2007). Reliability and validity of the Korean version of Kidney Disease Quality of Life instrument (KDQOL-SF). Tohoku J. Exp. Med..

[22] Han CW, Lee EJ, Iwaya T, Kataoka H, Kohzuki M (2004). Development of the Korean version of Short-Form 36-Item Health Survey: Health related QOL of healthy elderly people and elderly patients in Korea. Tohoku J. Exp. Med..

[23] Sweiss N, Sharma K (2014). Adiponectin effects on the kidney. Best Pract. Res. Clin. Endocrinol. Metab..

[24] Shibata R (2005). Adiponectin protects against myocardial ischemia-reperfusion injury through AMPK- and COX-2-dependent mechanisms. Nat. Med..

[25] Menon V (2006). Adiponectin and mortality in patients with chronic kidney disease. J. Am. Soc. Nephrol..

[26] Kollerits B, Fliser D, Heid IM, Ritz E, Kronenberg F (2007). Gender-specific association of adiponectin as a predictor of progression of chronic kidney disease: The Mild to Moderate Kidney Disease Study. Kidney Int..

[27] Perlman RL (2005). Quality of life in chronic kidney disease (CKD): A cross-sectional analysis in the Renal Research Institute-CKD study. Am. J. Kidney Dis..

[28] Kalantar-Zadeh K, Kopple JD, Block G, Humphreys MH (2001). Association among SF36 quality of life measures and nutrition, hospitalization, and mortality in hemodialysis. J. Am. Soc. Nephrol..

[29] Lopes AA (2007). Factors associated with health-related quality of life among hemodialysis patients in the DOPPS. Qual. Life Res..

[30] Bobin-Dubigeon C (2017). Leptin and adiponectin as new markers of undernutrition in cancer. Clin. Biochem..

[31] Dervisoglu E, Eraldemir C, Kalender B, Kir HM, Caglayan C (2008). Adipocytokines leptin and adiponectin, and measures of malnutrition-inflammation in chronic renal failure: Is there a relationship?. J. Ren. Nutr..

[32] Hara H (2018). Protein energy wasting and sarcopenia in dialysis patients. Contrib. Nephrol..

[33] Drechsler C, Krane V, Winkler K, Dekker FW, Wanner C (2009). Changes in adiponectin and the risk of sudden death, stroke, myocardial infarction, and mortality in hemodialysis patients. Kidney Int..

[34] Suh SH (2020). Chronic kidney disease attenuates the impact of obesity on quality of life. Sci. Rep..

[35] Pajvani UB (2003). Structure-function studies of the adipocyte-secreted hormone Acrp30/adiponectin. Implications fpr metabolic regulation and bioactivity. J. Biol. Chem..

[36] Pajvani UB (2004). Complex distribution, not absolute amount of adiponectin, correlates with thiazolidinedione-mediated improvement in insulin sensitivity. J. Biol. Chem..

[37] Aso Y (2009). Anemia is associated with an elevated serum level of high-molecular-weight adiponectin in patients with type 2 diabetes independently of renal dysfunction. Transl. Res..

[38] Gerson A (2004). Anemia and health-related quality of life in adolescents with chronic kidney disease. Am. J. Kidney Dis..

[39] van Haalen H, Jackson J, Spinowitz B, Milligan G, Moon R (2020). Impact of chronic kidney disease and anemia on health-related quality of life and work productivity: Analysis of multinational real-world data. BMC Nephrol..

[40] Eriksson D, Goldsmith D, Teitsson S, Jackson J, van Nooten F (2016). Cross-sectional survey in CKD patients across Europe describing the association between quality of life and anaemia. BMC Nephrol..

[41] Bloemer J (2018). Role of adiponectin in central nervous system disorders. Neural Plast..

[42] Diniz BS (2012). Reduced serum levels of adiponectin in elderly patients with major depression. J. Psychiatr. Res..

[43] Wędrychowicz A, Zając A, Pilecki M, Kościelniak B, Tomasik PJ (2014). Peptides from adipose tissue in mental disorders. World J. Psychiatry.

